# Use of *ex vitro* composite plants to study the interaction of cowpea (*Vigna unguiculata* L.) with the root parasitic angiosperm *Striga gesnerioides*

**DOI:** 10.1186/1746-4811-8-22

**Published:** 2012-06-28

**Authors:** Karolina E Mellor, Ava M Hoffman, Michael P Timko

**Affiliations:** 1 Department of Biology, University of Virginia, Gilmer Hall 044, Charlottesville, VA 22903, USA

**Keywords:** *Agrobacterium rhizogenes*, Composite plants, Hairy roots, Resistance, *Striga gesnerioides*, Witchweed

## Abstract

**Background:**

Cowpea (*Vigna unguiculata* L.) is an important grain and forage legume grown throughout sub-Saharan Africa primarily by subsistence farmers on poor, drought prone soils. Genetic improvement of the crop is being actively pursued and numerous functional genomics studies are underway aimed at characterizing gene controlling key agronomic characteristics for disease and pest resistances. Unfortunately, similar to other legumes, efficient plant transformation technology is a rate-limiting step in analysis of gene function in cowpea.

**Results:**

Here we describe an optimized protocol for the rapid generation of transformed hairy roots on ex vitro composite plants of cowpea using *Agrobacterium rhizogenes*. We further demonstrate the applicability of cowpea composite plants to study gene expression involved in the resistance response of the plant roots to attack by the root parasitic weed, *Striga gesnerioides*. The utility of the new system and critical parameters of the method are described and discussed herein.

**Conclusions:**

Cowpea composite plants offer a rapid alternative to methods requiring stable transformation and whole plant regeneration for studying gene expression in resistance or susceptibility responses to parasitic weeds. Their use can likely be readily adapted to look at the effects of both ectopic gene overexpression as well as gene knockdown of root associated defense responses and to the study of a broader range of root associated physiological and aphysiological processes including root growth and differentiation as well as interactions with other root pests, parasites, and symbionts.

## Background

Cowpea (*Vigna unguiculata* L. Walp.) is the most important grain legume grown in sub-Saharan Africa [1,2]. Approximately 12.5 million tons of cowpea grains are produced worldwide each year with a majority (over 64%) of the production taking place on low-input, subsistence farms in West and Central Africa [3]. Two characteristics contribute to its agronomic and economic importance. The plant is generally drought tolerant and can provide some yield even under harsh conditions, and it fixes nitrogen symbiotically thereby enhancing soil fertility especially when used in rotation with cereals [4,5]. Throughout the Sahel cowpea is referred to as “poor man’s meat” because of its high protein content (20-25%) and good nutritional value [6]. The pods and seeds are consumed at all stages of growth (e.g., green pods, fresh or dry seeds) and the young leaves are often used for soups and stews [7]. In addition to its value as human food, cowpea hay is an important source of animal fodder [8]. Improvement of cowpea as a multifunctional crop is a key breeding concern and significant efforts are currently aimed at its genetic improvement [2].

Like most crops, cowpea growth and grain yields are greatly reduced by a variety of biotic pests (e.g., bacterial, fungal, and viral diseases, insects, nematodes, and herbivores) and abiotic stresses (severe drought, salinity, and heat) [2]. Among the major biotic constraints is parasitism by *Striga gesnerioides* (L.) Walp. (Orobanchaceae) commonly referred to as witchweed. Witchweeds are noxious and persistent pests in farmers’ fields and yield losses due to *S. gesnerioides* parasitism are extensive in the Sudano-Sahelian belt of West and Central Africa [9]. Control of the parasite is difficult because it produces thousands of seeds per generation that remain in the seed bank for years and most of the damage to its host plant occurs prior to its emergence from the ground [10]. The damaging effects of *Striga* in this region are further compounded by poor soils and drought [11].

While most cowpea plants are susceptible to *Striga* parasitism, some local landraces and wild accessions have been identified that are resistant to the parasite, and in most reports resistance is a dominant characteristic, inherited in a monogenic manner [2,12]. Complicating the identification of *Striga*-resistant germplasm is the variable nature of the parasite with at least seven distinct races of *S. gesnerioides* (designated SG1 (Burkina Faso), SG2 (Mali), SG3 (Nigeria and Niger), SG4 (Benin), SG4z (Zakpota region of Benin), SG5 (Cameroon), and SG6 (Sénégal)) now identified throughout West Africa [13-15]. Analysis of several advanced populations segregating for resistance to one or more of the different races of *S. gesnerioides* has resulted in the genetic mapping of several race-specific resistance (R) genes within the cowpea genome and the development of molecular markers liked to these genes [16]. Using a positional cloning approach, Li and Timko [17] isolated and characterized a gene (designated *RSG3-301*) capable of conferring resistance to *S. gesnerioides* race 3 (SG3). *RSG3-301* encodes an R protein homolog containing a coiled-coil (CC) protein-protein interaction domain at the N-terminus, a nucleotide binding site (NBS), and a leucine-rich repeat domain at the C-terminus. Silencing of *RSG3-301* in the resistant cultivar B301 leads to susceptibility to race SG3, but does not affect resistance to other races of the parasite, underscoring the specificity of the resistance response [17].

Resistant cowpea genotypes exhibit two different response mechanisms to *Striga* attack. When challenged by a known race, cultivars carrying the appropriate race specific resistance gene exhibit a rapid and robust hypersensitive response typified by a browning and necrosis at the site of parasite attachment, and subsequent rapid death of the parasite within 3–4 days [15,18,19]. In host plants lacking the appropriate resistance gene, the parasite rapidly penetrates the host root cortex, forms connections to the host vascular system, swells to form a tubercle, and expands its cotyledons leading to subsequent above ground growth and flowering.

Like most legumes, the genetic transformation and regeneration of cowpea has proven to be difficult and challenging [20]. Cowpea is susceptible to genetic transformation by *Agrobacterium*, and a number of reports have appeared in the literature describing stably integrated and heritable transgenes following *A. tumefaciens*-mediated transformation [21-28]. However, *in vitro* regeneration of shots/seedlings from various seedling explants (including primary leaves [29], epicotyl [30], mature cotyledon [31], cotyledonary node [32,33] and nodal thin cell layer [34]) appears to be highly dependent on cultivar genotype with even the most promising genotypes giving very low regeneration frequency and numbers of regenerants. Coupled to transformation, the efficiency of recovery of fertile transgenic cowpea plants ranges from 0.1% to a 1-2% [25-28]. Given this limitation, traditional transgenic approaches for gene functional characterization in cowpea are difficult.

*Ex vitro* composite plants consist of a wild-type shoot with transgenic roots induced by transformation with *A. rhizogenes*[35-37]. The use of *ex vitro* composite plants has proven to be a successful alternative strategy for candidate gene analysis and genetic pathway dissection in plants where transformation and regeneration is difficult, time consuming and infrequent. The technique has been applied to the study of root nutrient uptake, hormone transport, interactions with root nodulating bacteria and mycorrhizal symbiotic, and parasitic nematodes [35,36,38]. Here, we present a protocol for the rapid development of transformed hairy roots on composite plants of cowpea using *A. rhizogenes* induced root formation. We further demonstrate the applicability of this approach to studying gene function involved in the interaction of cowpea with the root parasitic angiosperm, *S. gesnerioides*.

## Results

### An optimized protocol for the generation of *ex vitro* composite cowpea plants

**Figure 1:**
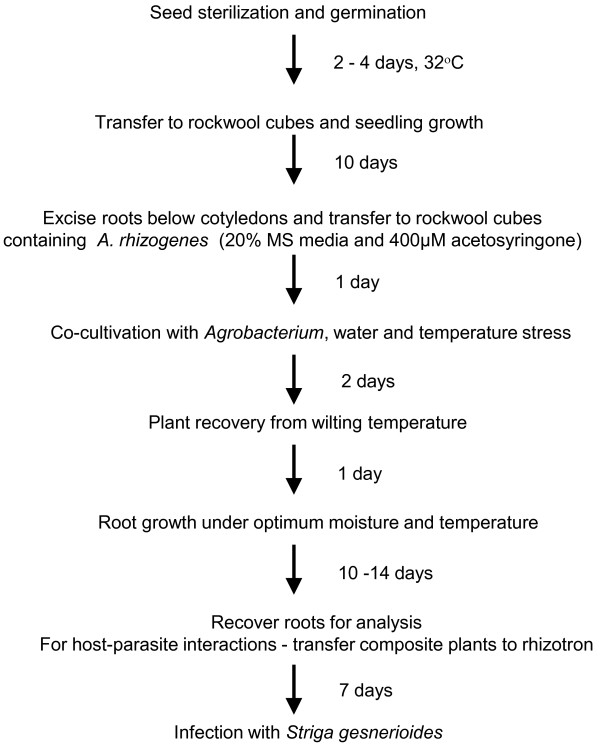
**Schematic representation of the time-line and key activities required for the generation of *ex vitro* composite cowpea plants.**

**Figure 2:**
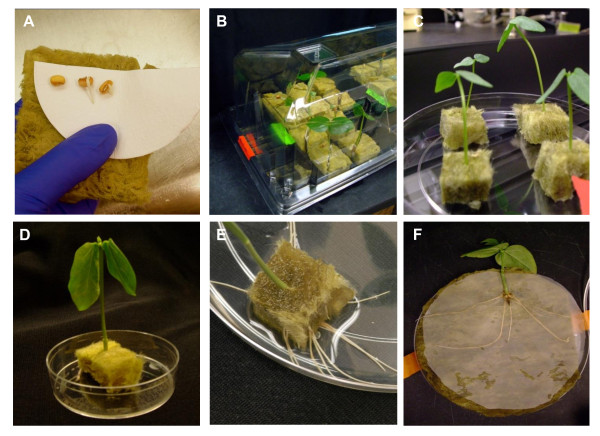
**Illustrations depicting the main steps for the production of *ex vitro* composite cowpea plants. A.** Pregerminated seeds are placed between two pieces of rockwool for further development. **B.** A 10 day old cowpea plant growing* in vitro* in rockwool growth chamber prior to *Agrobacterium rhizogenes* infection. **C.** Cowpea with a cut off root inserted into a rockwool cube saturated with *Agrobacterium rhizogenes* harboring pK7WG2D with GFP. **D.** Stressing infected cowpea by drying until leaf wilting. **E.** Transgenic roots growing out of the rockwool cube 12–14 days after infection with *A. rhizogenes*. **F.** Cowpea plant with transformed hairy roots in a Petri dish rhizotron prior to *Striga* infection.

The creation of *ex vitro* composite plants consisting of wild-type shoots and transgenic roots induced by transformation with *A. rhizogenes* has been described for several species [35-38]. Based on these prior studies we designed and optimized a protocol (Figure 1) that reliably gives a high yield of transformed roots suitable for downstream functional analysis. The general protocol is illustrated in Figure 2. Cowpea seeds are first disinfested to reduce contamination by extraneous fungal and bacterial sources and germinated on sterile moistened glass fiber filters at 32°C (Figure 2A). The seedlings are then transferred to moistened rockwool cubes and grown for 10 days in a humidified growth chamber at 30°C until the first trifoliate leaves have expanded (Figure 2B). Plants showing any signs of disease are discarded, and only vigorous, healthy looking cowpea plants are carried forward for the production of composite plants. Root tissues of healthy plants are excised below the cotyledons and placed in rockwool cubes moistened with MS media containing a diluted *A. rhizogenes* suspension (Figure 2C). The plants are co-cultivated with *Agrobacterium* for 2–3 days, during which time they are placed under water and temperature stress (Figure 2D). The plants are then allowed to recover from wilting and grown for 10–14 days until transgenic roots emerged from the rockwool cube (Figure 2E). At this point there is a mixture of transgenic and non-transgenic roots. Both can be used for studying various root associated functions. Here we have devised protocols suitable for the study of host-parasite interactions. For these studies the composite plants are transferred to a Petri dish rhizotron where they can be challenged with the parasitic angiosperm *S. gesnerioides* (Figure 2F).

At multiple points in the protocol we empirically determined the optimal conditions for recovery of composite cowpea plants. Since composite plants potentially generate a mixture of transgenic and non-transgenic roots, in order to optimize transformation efficiency (defined as the percentage of plants exhibiting one or more transgenic roots) and increasing the portion of transgenic versus untransformed roots, we used high level constitutive expression of green-fluorescent protein (GFP) as a biomarker for transformation. In these studies, plasmid pCambia1300-Gmubi3 [39], containing the soybean polyubiquitin 3 promoter fused to the coding region of GFP, was introduced into *A. rhizogenes* R1000 strain and used to determine the best conditions for cowpea transformation.

**Figure 3:**
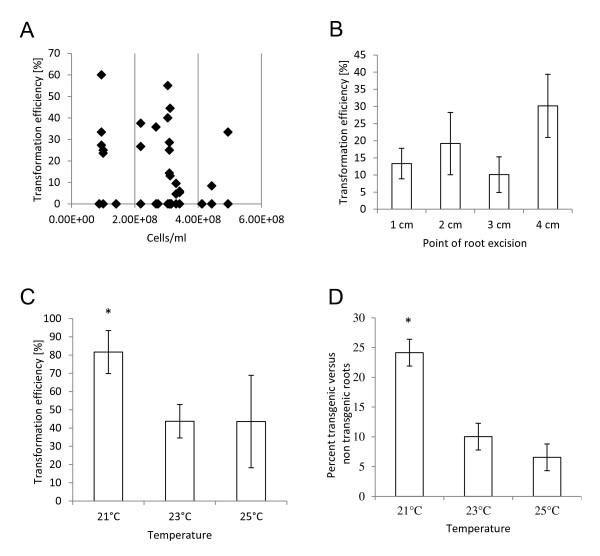
**Factors affecting transformation efficiency and recovery of composite plants. A.** Effects of multiplicity of infection during co-cultivation on the recovery of transgenic plants (transformation efficiency). **B.** Effects of location of root excision on transformation efficiency. Roots were cut at varied lengths (cm) below the cotyledon. **C.** Effect of temperature on transformation efficiency. Cowpea plants were co-cultivated with *A. rhizogenes* R1000 at the temperatures indicated. Number of transgenic roots was determined by presence of a GFP signal. Experiments were carried out in duplicate using up to 170 plants total. Values are means ± SE. Statistical significance was determined using one-way ANOVA. P < 0.05 is denoted by an asterisk. **D.** Percentage of newly formed transgenic roots on the positively transformed cowpea plant co-cultivated with *A. rhizogenes* at three different temperatures. Roots were assessed for GFP after emerging from a rockwool cube 12–14 days after transformation. The data show results from more than 1,700 roots total. Values are means ± SE. Statistical significance was determined using one-way ANOVA. P < 0.001 is denoted by an asterisk.

As shown in Figure 3 over a concentration range of 8 x 10^7^ cells/ml to 5 x 10^8^ cells/ml we did not observe a strong correlation between multiplicity of infection and transformation efficiency (Figure 3A). There was also no correlation between multiplicity of infection and proportion of transgenic to non-transgenic roots. We also found that the point of root excision (i.e., the distance from the point of cotyledon attachment to cutting) did not significantly influence transformation efficiency (Figure 3B). Increasing the root surface area for infection by cutting the stem at a 45^o^ angle rather that perpendicular to the long axis of the stem did not significantly alter transformation rates or recovery of transgenic roots. Since it is possible that the wound signal sensed by the *Agrobacterium* could be limiting, and to ensure maximal activation of virulence functions in the *Agrobacterium*, we increased the concentration of acetosyringone in the co-cultivation medium from 0.2 mM to 0.4 mM and this resulted in a small increase in transformation (data not shown).

Among the factors that significantly enhance transformation efficiency, we found that the temperature at which co-cultivation with *A. rhizogenes* was carried out and the length of the dehydration stress period imposed following co-cultivation were most important. As shown in Figure 3 plants grown at 21°C showed the highest transformation rate. Over 80% of plants infected with *Agrobacterium* harboring pCambia1300-Gmubi3 binary vector underwent a stable transformation (Figure 3C). Plant transformation efficiency was significantly lower when co-cultivated with *A. rhizogenes* at 23°C. Less than 40% of plants expressed GFP in their roots. Temperature also had an impact on the percentage of transgenic versus non-transgenic roots formed on the composite plants, with plants transformed at 21°C exhibiting a significantly higher percentage of stably transformed roots (~25% of total roots generated) compared with 23°C (~10% transgenic) and 25°C (5% transgenic), respectively (Figure 3D).

**Figure 4:**
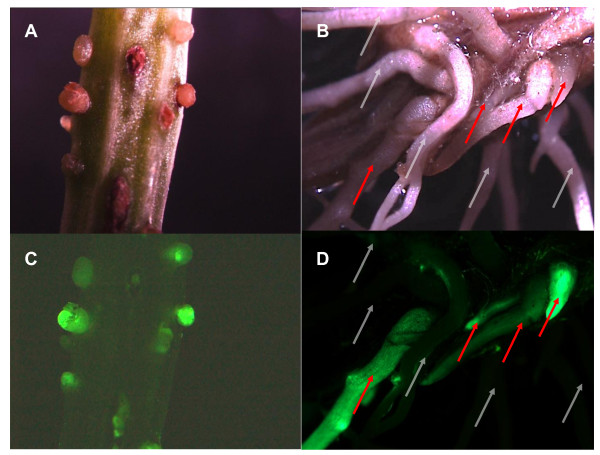
**Illustration of cowpea composite plants early and approximately two week after initiation of transgenic roots. A.** Light microscopic picture of early root initials following transformation of cowpea by *A. rhizogenes* containing pCambia1300-Gmubi3. **B.** Light microscopic picture of roots on cowpea approximately two weeks after transformation with *A. rhizogenes* containing pCambia1300-Gmubi3. **C.** Fluorescence microscopic picture corresponding to Panel A of early root initials following *A. rhizogenes* transformation of cowpea with pCambia1300-Gmubi3. **D.** Fluorescence microscopic picture corresponding to Panel B of roots on cowpea approximately two weeks after *A. rhizogenes* transformation of cowpea with pCambia1300-Gmubi3. In Panels B and D the gray arrows indicate non-transgenic roots and the red arrows indicate transgenic roots as judged by the absence or presence of GFP fluorescence, respectively.

**Figure 5:**
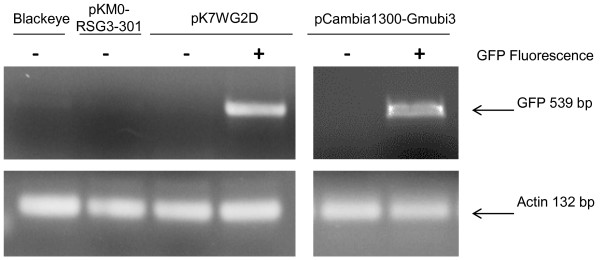
**Analysis of GFP transcript levels in roots of composite cowpea plants.** Transcript encoding GFP were measured in the roots of control Blackeye plants and in composite plants transformed with either pKM0-RSG-301 (vector with RSG3-301 minus GFP coding region), pK7WG2D (containing the prolD-GFP chimeric gene) and pCambia1300-Gmubi3 (containing the Gmubi3-GFP chimeric gene). PCR was carried out using gene specific primers directed to the GFP coding region. The predicted GFP amplification product is indicated; actin gene expression was used as control. The (+) indicates root tissues that gave a visual GFP fluorescence signal; (−) indicate root tissues that did not give a visual fluorescence signal.

As shown in Figure 4, it is possible to visually identify initiation of transgenic roots and the presence of both transformed and untransformed roots by appearance of a GFP signal. To confirm that the observed fluorescence was the result of stable transformation by a GFP transgens, the cowpea cultivar Blackeye was transformed with *A. rhizogenes* containing either pCambia1300-Gmubi3 or pK7WG2D (containing a prolD-GFP chimeric gene) and *GFP* transcript levels measured in pooled samples of fluorescent and non-fluorescent roots on composite plants. As shown in Figure 5, no GFP transcript is detectable in the roots of untransformed Blackeye plants or in Blackeye plants transformed with pKM0-RSG3-301 (a vector construct lacking GFP; described below). In contrast, when Blackeye plants were transformed with either pCambia1300-Gmubi3 or pK7WG2D, pooled transgenic roots from individual plants collected on the basis of a visible GFP signal gave a 539 bp PCR product indicative of GFP transcripts, whereas roots lacking a visible green fluorescence yielded no corresponding PCR product.

### Use of composite plants to study *striga*-host interactions

We have previously demonstrated that resistance in cowpea to parasitism by *S. gesnerioides* is conferred by monogenically inherited resistance genes that function in a race-specific manner [16,17]. The cowpea cultivar Blackeye is known to be susceptible to all races of *S. gesnerioides* identified in West Africa [15]. Having established conditions for the efficient generation of cowpea composite plants, we next explored whether our optimized protocol could be used for the study of root associated activities, in particular the interaction of cowpea cultivars with *S. gesnerioides*. To this end the cowpea *RSG3-301* gene, known to confer resistance to *S. gesnerioides* race SG3, was placed under the control of the constitutive CaMV 35S promoter in plasmid pK7WG2 and pK7WG2D yielding pKM0-RSG3-301 and pKMG-RSG3-301, respectively. pKM0-RSG3-301 allows for the overexpression of RSG3-301 alone, whereas pKMG-RSG3-301 expresses both RSG3-301 and GFP. The two plasmids were independently introduced into *A. rhizogenes* R1000 and used to generate composite plants using the optimized protocol described above. Composite plants were then used to determine whether (i) transformed roots of composite cowpea plants responded similarly to wild-type roots with respect to the resistance/susceptibility phenotype to attempted parasitism by *S. gesnerioides*, and (ii) whether introduction of a race-specific resistance gene (*RSG3-301*) into a susceptible cowpea genetic background (i.e., the Blackeye cultivar) confers race-specific resistance demonstrable in composite plants roots.

**Figure 6:**
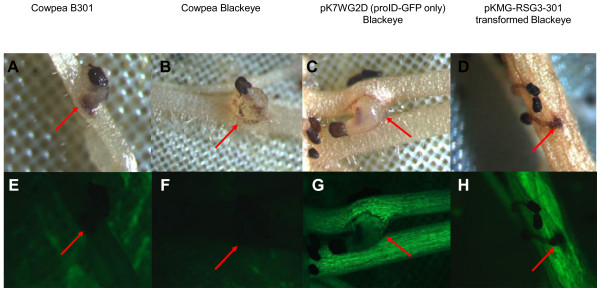
**Illustration of the response of roots on wild-type and composite cowpea plants to attempted parasitism by *Striga gesnerioides* race SG3.** Shown are photographs of representative cowpea root-*Striga* interactions. Pre-germinated *S. gesnerioides* race SG3 seedlings were placed in contact with cowpea roots and the interactions were analyzed at 8 days post inoculation. **A.** Light microscopic photograph and (**E**) associated fluorescence microscopic image of the hypersensitive response (HR) observed on the roots of resistant cowpea cultivar B301 when attached by *S. gesnerioides* race SG3. Note the browning and associated cell death in the cowpea root at the site of parasite infection, and browning of the attached parasite. **B.** Light microscopic photograph and (**F**) associated fluorescence microscopic image of showing the lack of an observed HR on the roots of susceptible cowpea cultivar Blackeye when attached by *S. gesnerioides* race SG3. Note that the parasite has formed xylem-xylem connections as evidenced by the presence of a tubercle. **C.** Light microscopic photograph and (**G**) associated fluorescence microscopic image showing tubercle growth of *S. gesnerioides* races SG3 on the transgenic roots of Blackeye cowpea transformed with the pK7WG2D empty vector construct. **D.** Light microscopic photograph and (**G**) associated fluorescence microscopic image of the hypersensitive response (HR) observed on the roots of Blackeye cowpea transformed with the pKMG-RSG3-301 resistance gene construct. Note the visible HR at the site of SG3 attachment similar to that observed in the interaction with the resistant cowpea cultivar B301. Arrows indicate host-parasite interaction.

**Figure 7:**
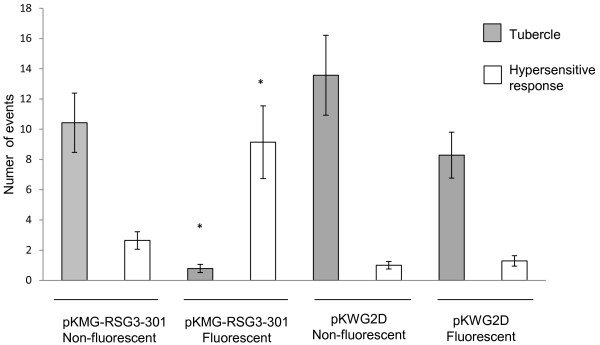
**Quantitative analysis of resistance response of transgenic and non-transgenic roots on composite cowpea plants to attempted parasitism by *S. gesnerioides* race SG3.** The number of parasite attachments showing either HR responses or tubercle growth were evaluated on the roots of composite cowpea plants generated by transformation with either pK7WG2D (which contains a prolD-GFP construct but lacks the *RSG3-301* resistance gene) or pKMG-RSG3-301 (which contains a prolD-GFP construct and a CaMV35S-RSG3-301 resistance gene construct). The number of events was counted 8 dpi with *S. gesnerioides* race SG3. Data show the results of counts from 14 independent plants. Error bars indicate the standard error (SE). Events were counted on roots determined visually to be both transgenic and expressing GFP or non-transgenic and non-GFP fluorescent. Statistical significance was determined using one-way ANOVA. P < 0.001 is denoted by an asterisk.

As shown in Figure 6, when roots of the cowpea cultivar Blackeye are attacked by *S. gesnerioides* race SG3, no visible resistance response is observed, and within 5–7 days post-attachment of the parasite a small parasite tubercle begins to develop (Figure 6B and 6F). In contrast, when a cowpea cultivar such as B301 which contains the *RSG3-301* resistance gene is similarly challenged within the same time frame come a visible hypersensitive response develops at the site of parasite attachment leading to browning, apoptosis at the point of parasite attachment and eventual death of the attached *Striga* seedling (Figure 6A and 6E). Composite plants generated from cowpea cultivar Blackeye transformed with *pK7WG2D* vector (mock transformation) generate a combination of both transformed and untransformed roots. When challenged with *S*. *gesnerioides* SG3 both transformed and untransformed roots respond similarly, failing to mount a resistance response and allowing tubercle formation similar to what is observed in wild-type (non-composite) Blackeye (Figure 6C and 6G). In contrast, transgenic roots on composite plants generated by transformation with *A. rhizogenes* containing pKMG-RSG3-301 exhibited a dramatically different response (Figure 6D and 6H). In this case, the pKMG-RSG3-301 transformed roots mount a HR response similar to that observed in the resistant cowpea cultivar B301. In order to quantify this effect we compared the proportion of HR events and amount of tubercle formation on transgenic and non-transgenic roots of Blackeye composite plants generated using either pK7WG2D (prolD-GFP, no *RSG3-301* gene) or pKMG-RSG3-301 (prolD-GFP, overexpressed *RSG3-301* gene). The results are shown in Figure 7. Both transgenic and non-transgenic roots of composite plants generated using pK7WG2D showed high levels of tubercle formation and little or no HR response when subjected to *Striga* parasitism consistent with the fact that the prolD-GFP chimeric gene alone has no effect on the resistance or susceptibility response of the composite plant. In contrast, there was a significant (p < 0.001) increase in the number of HR events on the transgenic roots generated by transformation of Blackeye with pKMG-RSG3-301 (prolD-GFP, overexpressed *RSG3-301* gene) following challenge by *S. gesnerioides* SG3. In this case, overexpression of the *RSG3-301* gene conferred a typical resistance response similar to that observed in the resistant cultivar B301. In contrast, the response to *Striga* attack on non-transformed roots of composite Blackeye plants generated with pKMG-RSG3-301 were similar to that of wild type Blackeye or Blackeye transformed with pK7WG2D with multiple parasite tubercles and few HR responses.

**Figure 8:**
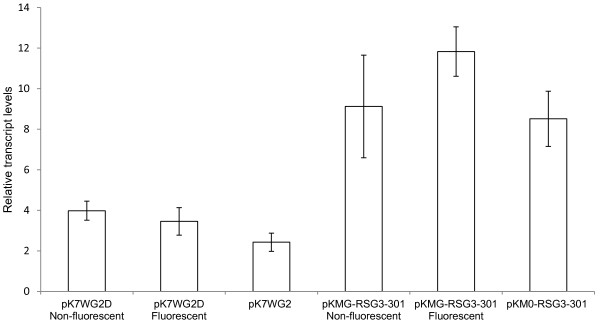
**qRT-PCR analysis *RSG3-301* transcript levels in transgenic and non-transgenic roots of cowpea composite plants.** Transcripts encoding the *RSG3-301* resistance gene were determined by qRT-PCR using total RNA isolated from transgenic and non-transgenic roots of composite Blackeye plants generated using the following constructs: pK7WG2D (prolD-GFP, no *RSG3-301* gene), pK7WG2 (no GFP, no *RSG3-301* gene), pKMG-RSG3-301 (prolD-GFP, overexpressed *RSG3-301* gene) and pKM0-RSG-301 (no GFP, overexpressed *RSG3-301* gene). Relative *RSG3-301* transcript levels were obtained from calibrating its threshold cycles relative to control actin transcripts as described in the Materials and Methods. Means and standard errors (SE) based on from the 5 independent biological replicates are given.

To demonstrate that increased levels of *RSG3-301* transcripts were present in the transformed roots and its expression correlates with both GFP (biomarker for transformation) and the resistance phenotype, qRT-PCR was performed using RNA isolated from transformed and untransformed roots of the composite plants generated using either pK7WG2D (prolD-GFP, no *RSG3-301* gene), pK7WG2 (no GFP, no *RSG3-301* gene), pKMG-RSG3-301 (prolD-GFP, overexpressed *RSG3-301* gene) and pKM0-RSG-301 (no GFP, overexpressed *RSG3-301* gene) (Figure 8, Table 1). The result shows that, as expected, *RSG3-301* transcripts are highly abundant in roots scored as transgenic by virtue of the presence of a positive GFP fluorescence in pKMG-RSG3-301 transformed plants. Somewhat unexpectedly, *RSG3-301* transcripts were detected in pooled samples of non-fluorescent (presumed non-transgenic) roots of pKMG-RSG3-301 transformed plants. The levels of RSG3-301 transcripts in the non-fluorescent-root samples from pKMG-RSG3-301 transformed plants were highly variable. It is possible that prolD-GFP transgene expression was lost or very low in these samples leading to their being miscalled as non-transgenic. As expected, *RSG3-301* transcripts were found in the pooled samples of roots from pKM0-RSG-301 transformed plants. In this case, no GFP biomarker is present so it was not possible to distinguish transformed and untransformed roots on the plant which leads to a greater variability between samples. *RSG3-301* was not significantly detected in either transgenic roots generated using pK7WG2D (fluorescent, non-fluorescent), or the roots on pK7WG2 transformed Blackeye.

**Table 1:** **Tabular data of relative *RSG3-301* transcript levels in transgenic and non-transgenic roots of cowpea composite plants**

**Sample name**	**Sample 1**	**Sample 2**	**Sample 3**	**Sample 4**	**Sample 5**	**Average relative transcript levels**	**±SE**
pK7WG2D	4.59	5.28	4.14	3.25	2.64	3.98	0.47
Non-fluorescent							
pK7WG2D	3.82	1.61	4.60	2.17	5.07	3.45	0.67
Fluorescent							
pK7WG2	4.00	2.07	2.14	1.27	2.64	2.43	0.45
							
pKMG-RSG3-301	3.07	10.56	16.95	10.93	4.12	9.12	2.53
Non-fluorescent							
pKMG-RSG3-301	12.85	9.51	8.88	15.63	12.27	11.83	1.22
Fluorescent							
pKM0-RSG3-301	8.57	11.71	4.14	7.21	10.93	8.51	1.36

## Discussion and conclusions

The recalcitrance of cowpea to efficient transformation and regeneration has been a bottleneck in functional genomics analysis and bioengineering for genetic improvement of the crop. The current study addresses one of these fundamental limitations by providing a fast and relatively easy method to rapidly generate and evaluate multiple independent transformants for screening gene expression associated with root localized plant functions. The transformation efficiency we have achieve (up to 80% plants with at least one transgenic root; and 25% of all roots formed being transgenic) with our optimized protocol is comparable to that obtained by Collier et al [36] for soybean who reported between 2 and 4 transformed roots per transformed plant with a transformation efficiency of ~ 80%. It is also similar to that reported for *MedicagoPhaseolus* and pea [35,37,38] although direct comparisons are more difficult because of the manner in which transformation rates were measured. Nonetheless, the recovery of transgenic events we achieve are significantly higher than the efficiency of recovery of fertile transgenic cowpea plants using traditional *A. tumefaciens* –based transformation and regeneration which range from 0.1% to a 1-2% [25-28]. It should be noted that the limitations of the composite system are that although these are stable transformations they do not allow recovery of stable fertile offspring and therefore analysis is constrained to only the initial generation.

The great advantage of the composite system is the utility for studying root associated parameters such as parasitism by weedy parasitic angiosperm and other root pests and pathogens. In this regard, the use of composite plants make it possible to look at the effects of both ectopic gene overexpression as well as gene knockdown on root associated defense responses. As demonstrated in our above studies, the composite plant system was capable of recapitulating what is found in non-transgenic plants with regard to the resistance or susceptibility response to attempted *Striga* parasitism. Overexpression of a race-specific resistance gene (e.g., *RSG3-301*) in a susceptible genotype resulted in acquisition of the resistance phenotype similar to what is observed in a naturally occurring resistant cultivar such as B301. This observation indicates that the process of transformation does not fundamentally change the nature of the signal transduction apparatus involved in defense in the transgenic roots. This might have been the case since the introduction of ROL genes by *A. rhizogenes* and generation of the hairy-root phenotype is known to be associated with alterations in phytohormonal concentrations [40]. Clearly, this is not significantly impacting the defense processes (or lack thereof) in the plant. This result opens up the potential for using the composite plant approach to evaluate various candidate genes that might be involved in cowpea resistance or susceptibility to *S. gesnerioides*, as well as other root parasitic angiosperms of economic significance such as *Alectra vogelii*. The utility as noted by Collier et al [36] lies in the fact that candidate gene constructs identified by screening in composite plants can be moved immediately without further manipulation to *A. tumefaciens* for the production of stable transgenic plants. In the case of cowpea, where the efficiency of traditional *A. tumefaciens* –based transformation and regeneration is low, this would result in a significant economy of time and effort.

The cowpea composite plant system also has applicability to studying gene expression in other physiological processes such as root development, growth and differentiation, and aphysiological conditions such as bacterial, fungal or viral induced diseases and interactions of roots with parasitic nematodes, nodulation by symbiotic bacteria, and colonization by mychorrizal fungi.

## Materials and methods

### Plant materials and growth

Descriptions of the cowpea accession used in this study can be found in [17]. B301 is a *Striga* multi-race resistant cultivar from Botswana; Blackeye is a *Striga* susceptible commercial variety. Seeds of the cowpea cultivars B301 and Blackeye were surface-sterilized with 1% Metricide (Metrex, Romulus, MI) for 5 min [41]. They were then placed between two sheets of moist glass fiber filter paper (GF/A Whatman; Piscataway, NJ), held between two blocks of moistened rockwool (Grodan Inc., Milton, ON) for 10 days until leaves have fully developed. Plants were grown at 30°C in a growth chamber to ensure moist environment. The chamber was covered with a transparent dome which light passage.

### Vector construction and generation of *Agrobacterium rhizogenes* strains

The structure of the pCambia1300-Gmubi3 vector has been previously reported [39] and contains green fluorescent protein (GFP) coding region under the control of the soybean polyubiquitin 3 promoter. Gateway compatible destination vectors pK7WG2 and pK7WG2D [42] were obtained commercially (VIB, Brussels, Belgium). For overexpression of the cowpea *RSG3-301* resistance gene [17], the *RSG3-301* coding region was mobilized into the pENTR/D-TOPO intermediate vector (Invitrogen, Grand Island, NY) and pENTR/D-TOPO-RSG3-301 recombined with pK7WG2D using the LR recombination reaction in the Gateway LR Clonase Enzyme mix (Invitrogen, Grand Island, NY). The resulting plasmid is designated, pKMG-RSG3-301. The *RSG3-301* coding region in pENTR/D-TOPO-RSG3-301 was similarly recombined with pK7WG2 generating pKM0-RSG3-301. The relevant portions of pKMG-RSG3-301 and pKM0-RSG3-301 were sequenced to confirm the integrity of the introduced gene.

The various plasmids (pCambia1300-Gmubi3, pKMG-RSG3-301 and pKM0-RSG3-301) were transformed into *Agrobacterium rhizogenes* R1000 competent cells by electroporation and transformed colonies were selected by growth for two days on solid YEB medium containing 0.1 mM KAN. Independent positive transformants were picked and inoculated into liquid YEB medium containing 0.1 mM KAN and grown at 28°C until saturation. Cells were collected and plasmid DNA was prepared using the GeneJET Plasmid Miniprep Kit (Thermo Scientific, Glen Burnie, MD). The presence of the introduced plasmid into the Ti plasmid was verified by restriction digestion using BsrG1 enzyme (New England Biolabs, Ipswitch, MI). Colonies of Agrobacteria confirmed to be transformed with the various constructs were selected, grown in liquid culture, and stored at −80°C in 50% glycerol solution until ready to use.

### Plant transformation

Two days prior to plant transformation, *A. rhizogenes* carrying pKMG-RSG3-301, pKM0-RSG-301, pK7WG2D and pK7WG2 plasmids were thawed and streaked out on solid YEB medium containing 0.1 mM (50 mg/l) KAN, 0.4 mM acetosyringone and grown at 28°C. Individual colonies were picked and inoculated into 10 ml liquid YEB media with antibiotic and supplement as mentioned above and grown overnight until OD_600_ = 0.8 was reached (OD_600_ of 1.0 = 5 x 10^8^ cells/ml).

On the day of plant transformation, bacterial suspension was spun down at 3,500 rpm and resuspended in 15 ml 20% MS media with vitamins (Caisson labs, Logan, UT) supplemented with 0.4 mM acetosyringone. Bacteria were grown for another several hours until OD_600_ = 0.8 was reached and then 6 ml of the bacterial suspension was pipetted onto a 1 cm^3^ rockwool cube (Grodan Inc., Milton, ON) so that each cube was saturated.

Ten day-old cowpea plants were taken out of growth chamber, and the roots excised approximately 3 cm below the cotyledons using a sterile razor blade. The plant was then inserted into the bacteria-saturated rockwool cube and placed into a darkened growth chamber at 21°C to ensure high transformation efficiency. On the second day, a lid from the growth chamber was removed which allowed plants to dry. Applying drought stress appeared a crucial factor for increasing amount of transformed roots. Plants were rewatered after leaves became obviously wilted. This procedure was repeated two days in the row. On the fourth day plants were transferred into a 22°C growth room and allowed to grow in cubes. First transformed roots emerged from the cube 12–14 days after transformation. Plants were then taken from growth room and rockwool surrounding emerging roots was gently removed using forceps. Cowpea was then transferred to 24 cm x 24 cm x 3 cm growth chamber containing rockwool with a 100 μm mesh separating the cowpea roots from the rockwool [17]. Cowpea seedlings were grown for another 7 days in a controlled environment growth room under a 12 H light–dark photoperiod at 30°C. After a week, plants infected with pKMG-RSG3-301 were tested for GFP present in their roots using Zeiss Stereo Discovery V20 with Axciocam MRc High resolution camera. Several roots (transformed and non-transformed) were then collected and stored in −80°C for qRT-PCR to assess *RSG3-301* transcript levels in the tissue. Due to the lack of GFP, plants infected with pKM0-RSG3-301 were only collected for qRT-PCR.

### Assessing *Striga* – host interactions

*S. gesnerioides* is a federally regulated noxious weed. All work involving viable *S. gesnerioides* seeds, developing parasites, and all analysis of host-parasite interactions, were performed in our APHIS approved quarantine facility at UVA (Facility Number 669; Permit No. P526P-11-03310). Seeds of the various *S. gesnerioides* races were collected in the field as reported previously [15]. Seeds of *S. gesnerioides* race SG3 were surface sterilized, pre-conditioned for 9 days [15], and germination triggered using root exudates from cowpea cultivar B301. Pre-germinated seeds (50 mg equivalent to ~7500-10,000 seeds, with an average germination of 75% at time of infestation) were gently transferred on developed cowpea roots (pKMG-RSG3-301 fluorescent and non-fluorescent; pK7WG2D fluorescent and non-fluorescent) using a paint brush. Growth chambers were closed and left for another 8 days for infection to occur. After 8 dpi, infection rate was assessed. Number of events (tubercle formation and hypersensitive response) was counted on plants infected with pKMG-RSG3-301 for both fluorescent and non-fluorescent roots as well as plants infected with pK7WG2D. Number obtained from 14 individual plants per treatment were averaged and presented ± SE.

### Verification of RSG3-301 transcript levels using qRT-PCR

qRT-PCR was used to validate *RSG3-301* transcript levels of RNA samples prepared from Blackeye root tissues subject to the various treatments described above. Total RNA was extracted as described in [43] with minor modifications and quantified using a Nanodrop instrument (Thermo Scientific, Wilmington, DE). cDNA was synthesized from 2 μg of DNAse- treated (Roche, Indianapolis, IN) total RNA using an Invitrogen Thermoscript Kit (Invitrogen, Carlsbad, CA) according to manufacturer’s instructions. The reaction was carried out in triplicate.

For qRT-PCR reactions, gene-specific primers were designed against the *RSG3-301* gene coding region [17] that give a 147 bp amplicon and an actin reference gene (XM_003521168; GI: 356504867) giving a 132 bp amplicon. The primers used are as follows: RSG3-301-F: 5′- AGTAAGGGATGTTGGAAGCAA -3′and RSG3-301-R: 5′- AATTACATCAGACTCGGGAAT -3′); Actin-F: 5′- CGAGCAGGAATTGGAAAC-3′and Actin-R: 5′-ATCATGGATGGCTGGAAC-3′. Amplification reactions (25 μl) containing 15 ng of cDNA were carried out in Multiplate PCR 96-well plate formats (Bio-Rad, Hercules, CA), using the iQ SYBR Green supermix kit (Bio-Rad, Hercules, CA) in an iCycler Optical Module PCR instrument according to the manufacturer’s instructions quantification of relative transcript abundance was performed described by Schmittgen and Livak [44] by calibrating threshold cycles of the *RSG3-301* transcripts with that of the actin reference gene. The equation 2^(−ΔΔCq)^ was used to calculated relative expression (difference in concentration between samples based on normalization with a reference gene), in which C_q_ is the cycle number of the threshold point at which the fluorescence is detectable. A total of 5 independent biological replicates were used and mean and standard error (SE) were determined.

## Abbreviations

dpi, days post- inoculation; GFP, Green fluorescent protein; HR, Hypersensitive response; KAN, Kanamycin; LB, Luria bertoni; MS, Murashige and skoog; OD, Optical density; qRT-PCR, quantitative reverse transcriptase polymerase chain reaction; rpm, Revolutions per minute; YEB, Yeast extract and beef.

## Authors’ contributions

MPT directed the project, MPT and KEM designed the experiments, and KEM and AH carried out the experiments. MPT and KEM wrote the manuscript. All authors have read and approved the final manuscript.

## Competing interests

The authors declare that they have no competing interests.
